# Telling the Time with a Broken Clock: Quantifying Circadian Disruption in Animal Models

**DOI:** 10.3390/biology8010018

**Published:** 2019-03-21

**Authors:** Laurence A. Brown, Angus S. Fisk, Carina A. Pothecary, Stuart N. Peirson

**Affiliations:** Sleep and Circadian Neuroscience Institute (SCNi), Nuffield Department of Clinical Neurosciences, Oxford Molecular Pathology Institute, Sir William Dunn School of Pathology, South Parks Road, Oxford OX1 3RE, UK; laurence.brown@ndcn.ox.ac.uk (L.A.B.); angus.fisk@bnc.ox.ac.uk (A.S.F.); carina.pothecary@ndcn.ox.ac.uk (C.A.P.)

**Keywords:** activity bouts, data analysis, entrainment, fragmentation, periodogram

## Abstract

Circadian rhythms are approximately 24 h cycles in physiology and behaviour that enable organisms to anticipate predictable rhythmic changes in their environment. These rhythms are a hallmark of normal healthy physiology, and disruption of circadian rhythms has implications for cognitive, metabolic, cardiovascular and immune function. Circadian disruption is of increasing concern, and may occur as a result of the pressures of our modern 24/7 society—including artificial light exposure, shift-work and jet-lag. In addition, circadian disruption is a common comorbidity in many different conditions, ranging from aging to neurological disorders. A key feature of circadian disruption is the breakdown of robust, reproducible rhythms with increasing fragmentation between activity and rest. Circadian researchers have developed a range of methods for estimating the period of time series, typically based upon periodogram analysis. However, the methods used to quantify circadian disruption across the literature are not consistent. Here we describe a range of different measures that have been used to measure circadian disruption, with a particular focus on laboratory rodent data. These methods include periodogram power, variability in activity onset, light phase activity, activity bouts, interdaily stability, intradaily variability and relative amplitude. The strengths and limitations of these methods are described, as well as their normal ranges and interrelationships. Whilst there is an increasing appreciation of circadian disruption as both a risk to health and a potential therapeutic target, greater consistency in the quantification of disrupted rhythms is needed.

## 1. Circadian Rhythms

Circadian rhythms are ~24 h cycles of physiology and behaviour that occur in virtually all organisms from bacteria to man. These rhythms are generated by an internal biological clock and persist even in isolation from any external environmental cues. In mammals, the master circadian clock is located in the hypothalamic suprachiasmatic nuclei (SCN), and is based upon an intracellular transcriptional-translational feedback loop (TTFL), comprised of a number of core clock genes [1]. Output from the SCN in turn coordinates rhythms of physiology and behaviour, as well as synchronising peripheral clocks found in tissues and organs throughout the body [2]. As a result, the circadian system can be regarded as an integrated network of clocks, responding to neural, hormonal and behavioural signals to ensure an appropriate phase relationship with each other as well as the external world.

A clock is of no use unless it can be set to local time. In mammals, the primary time cue (zeitgeber) is light detected by the eye. The retinal photoreceptors mediating this response include the rods and cones involved in vision, as well as the recently identified photosensitive retinal ganglion cells (pRGCs) expressing the blue-light sensitive pigment melanopsin [3,4,5]. This light information is used to shift the molecular clock in the SCN, enabling the internal circadian system to be synchronised (entrained) to the external light/dark (LD) cycle [6].

## 2. Measuring Circadian Rhythms

**Figure 1:**
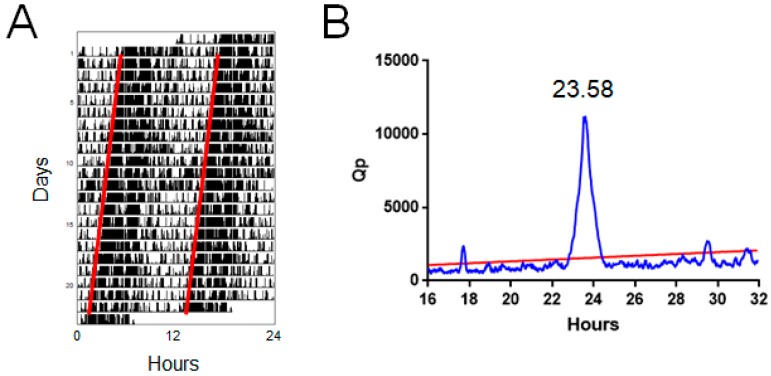
Standard measures of circadian activity. (**A**) Double-plotted actogram showing activity of a wildtype C57BL/6 mouse in constant darkness. The period of activity is slightly below 24 h, resulting in a small daily advance in activity onset (indicated by red lines). (**B**) Chi-square periodogram derived from A, showing a dominant period of 23.58 h. Sample data from Brown et al., 2016 [16] measured using PIR sensors.

In humans, circadian rhythms in activity are typically measured using calibrated wrist-worn accelerometers. By contrast, the activity of laboratory animals is typically measured using home cage running wheels [7,8,9,10]. In addition, infrared-beam break and passive infrared (PIR) sensors are also used. Circadian data are typically double plotted as actograms, showing activity across multiple days (Figure 1A). However, by itself the ability to measure physiology and behaviour over multiple days does not necessarily make it useful. The appropriate analysis of such data is essential to identify specific features of this data that are biologically informative. To this end, the circadian field has developed standard methods for analysing circadian rhythms. This primarily includes methods to detect recurring features in the data, enabling the period length of activity cycles to be determined. Under entrained conditions, this period will normally be determined by environmental zeitgebers. For example, a mouse housed under a 24 h LD cycle will display a 24 h period. Typically, only under constant conditions will the endogenous circadian period be evident, which in mice is typically ~23.5 h in constant dark [6,9,11]. It should be noted that the endogenous period can be manifested in the presence of a zeitgeber, for example under conditions outside the range of entrainment, such as an artificially short 22 h day [12]. This can give rise to several simultaneous periodicities. Furthermore, in nocturnal rodents such as mice, the endogenous circadian period under constant light conditions lengthens with increasing light intensity according to Aschoff’s rule [13].

A range of different methods are used to determine the underlying period in biological time series. Three of the most commonly used are the Enright periodogram, Fourier analysis and the Lomb-Scargle periodogram. In addition, activity onset is also frequently used to characterise phase shifts in rhythms in response to environmental zeitgebers. These different methods are briefly described below. However, for a discussion of the relative strengths and limitations of these methods the reader is referred to specific studies that have compared different methods of period estimation [14,15].

### 2.1. Enright Periodogram

The Enright periodogram is perhaps the most widely used method for circadian period estimation [17]. This approach involves systematically varying the timescale (modulo) on which circadian data are plotted to provide a quotient of variance across the different timescales. The largest value is obtained when modulo corresponds to the period in the data, resulting in the minimal variance. A common implementation of this approach is the chi-square periodogram which provide a ratio of variances termed Q_P_. Data are typically plotted as a periodogram of period in hours vs. Q_P_ (Figure 1B), where a significant period in the dataset can be detected by a Q_P_ value exceeding the expected background value based upon chi-square distribution [18]. 

### 2.2. Fourier Analysis

Fourier analysis is based upon the fact that any time series can be approximated by a series of sine and cosine waves of differing period, amplitude and phase. Using this approach, any complex time series can be decomposed into a series of simple waveforms, with coefficients determined based upon goodness of fit to the data. These spectral analysis methods are widely used to study biological time series [19]. One such implementation of Fourier analysis, the Fast Fourier Transform Non-Linear Least Squares (FFT-NLLS) method has been widely used to estimate the dominant circadian period within a dataset [20,21]. However, it should be noted that as the frequencies for which coefficients are determined are based upon 1/N, 2/N, etc.; (where N is the number of data points), frequencies around the circadian range may be limited. As such, the temporal resolution for circadian data is often not very high [14]. By contrast, Fourier methods may provide valuable information regarding ultradian rhythms within a dataset.

### 2.3. Lomb-Scargle Periodogram

The Lomb-Scargle periodogram is an adaptation of Fourier analysis to unequally spaced time series [22]. This method can adapt for missing values or irregular data, and has been shown to be just as effective as the chi-square periodogram for circadian rhythm analysis [23,24].

### 2.4. Activity Onset

As well as periodogram analysis, the timing of activity onset is also frequently used in circadian research. Linear regression based upon activity onset can be used to estimate circadian period [14]. However, it is more commonly used to study phase shifting responses to light and other zeitgebers [7]. Exposure to light in the early part of the dark phase results in a delay in activity onset the following day, whereas exposure to light late in the dark phase produces phase advances. Plotting the phase shifting response to light against time of day provides a phase response curve. In addition, phase shift magnitude can be measured over different light intensities to provide an irradiance response curve (IRC)—similar to a drug dose-response curve. Multiple IRCs can be generated to different wavelengths of light to produce an action spectrum, which describes the spectral sensitivity of any biological response to light and providing valuable information regarding the photopigment mediating the response [11]. Action spectra have played a key role in characterising the photoreceptors involved in non-visual responses to light in both humans and mice [25,26,27,28].

## 3. Circadian Disruption

Circadian rhythms are an important feature of normal healthy physiology, and circadian disruption is widely recognised to be detrimental to health. As well as disrupting physiological processes such as sleep, disturbances of the circadian system have been shown to contribute to impaired cognitive performance, metabolic and cardiovascular disorders and even the risk of developing cancer [29,30,31,32,33]. Circadian disruption may occur as a result of environmental conditions, particularly as a result of modern societal challenges to our 24 h physiology. This includes shift work, jet-lag and artificial light exposure which result in a misalignment of our circadian physiology with the external world. In addition, circadian disruption is a common feature of many different conditions [34], including—but not limited to—aging [35,36], metabolic disease [37], cardiovascular disease [38], cancer [39] and neurological disorders [31,33,36,40]. The causal relationship between such conditions and circadian disruption is unclear, but this comorbidity may contribute to disease burden or worsen existing symptoms. As such, the circadian system provides a valuable target for the treatment of a broad range of disorders, which may help alleviate disease burden as well as improving quality of life [41]. 

### Forms of Circadian Disruption

Despite its widespread use, the term ‘circadian disruption’ is largely an umbrella term for many different types of specific perturbations of circadian physiology [42]. This includes ‘misalignment’—when two or more rhythms adopt an abnormal phase relationship, and ‘desynchrony’—when two or more rhythms exhibit a different period. Furthermore, misalignment and desynchrony can refer to rhythms that are internal (e.g., central vs. peripheral clocks) or internal vs. external (e.g., central vs. external LD cycle). In addition, ‘chronodisruption’ is also used, typically to describe the mismatch between an individual’s biological night and work hours [43]. Finally, ‘social jetlag’ is also often used to describe the everyday challenge to the circadian system imposed by work and social commitments. Social jetlag is defined as the difference in sleep timing between work and free days, providing a useful metric of circadian misalignment [44]. 

## 4. Measuring Circadian Disruption

Different types of circadian disruption will produce characteristic changes in activity patterns. For example, circadian misalignment will result in changes in the onset and phasing of activity in relationship to the LD cycle [45]. By contrast, internal desynchrony—as observed under non-24 h T-cycles, may result in two or more rhythms with different periodicity [46]. Chronodisruption is expected to produce increased activity in the normal inactive phase (increased light phase activity in nocturnal rodents), with a reduction in the relative amplitude between activity and rest. Finally, social jetlag will result in changing activity onset between ‘work’ and ‘free’ days, though due to its relationship to human social activity such paradigms are rarely used in animal studies. In addition to these characteristic changes in activity observed under different circadian disruption paradigms, some consistent changes are observed across a wide range of different conditions. Whilst circadian period is typically unchanged when compared with normal healthy rhythms, activity rhythms become increasingly fragmented and variable. As such, the standard analytical tools used in circadian biology—optimised to detect changes in period—are not particularly suited for measuring circadian disruption. 

**Figure 2:**
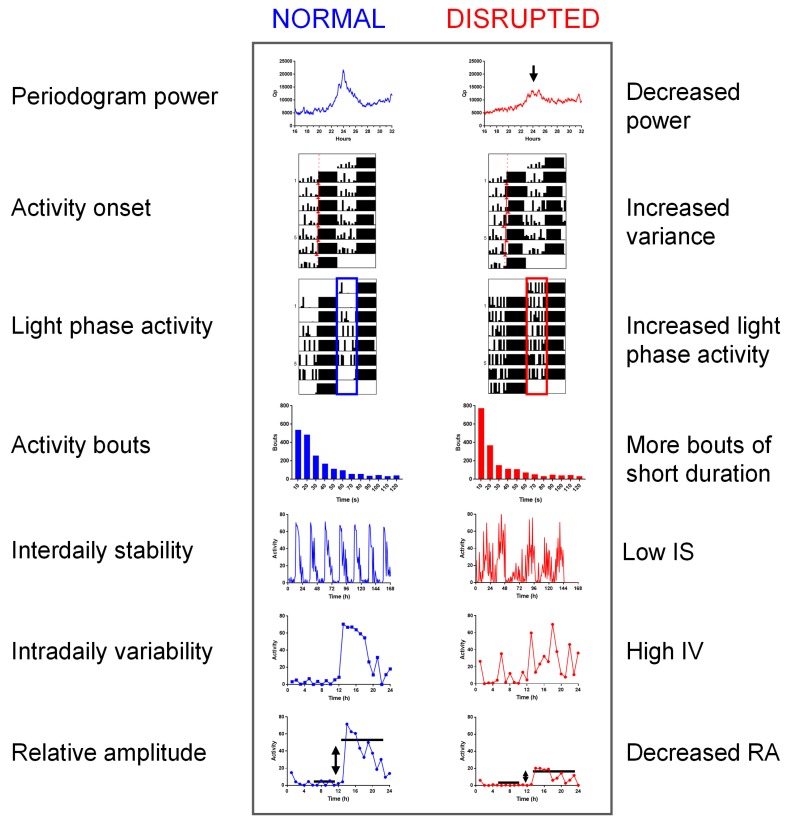
Commonly used measures of circadian disruption. Images of each parameter are shown for normal healthy animals (blue), along with representative changes that would be expected with circadian disruption (red). See text for details.

Throughout the literature a range of approaches have been used to assess circadian disruption. These methods range from simple visual inspection of actograms to metrics such as periodogram power, variability in activity onset, light phase activity, activity bouts, interdaily stability, intradaily variability and relative amplitude. These are described below in detail with reference to different forms of circadian disruption. In addition, we also use specific examples from genetic models relevant to neuropsychiatric disease studied in the author’s lab. These include the blind-drunk (*Bdr*) mutant, a mutation in the synaptosomal-associated protein (Snap)-25, which results in impaired synaptic exocytosis [47]; and metabotropic glutamate receptor 2 and 3 double knockout mice (*Grm2/3^−/−^*), which lack presynaptic inhibitory autoreceptors which have been implicated in schizophrenia [48]. We go on to discuss how these measures of circadian disruption are derived as well as providing additional detail relating to their strengths and limitations. Schematic examples of healthy versus disrupted rhythms are shown for each parameter in Figure 2 and normal ranges are shown in Table 1. The following discussion is based upon the study of circadian disruption in laboratory animal models (rats and mice), but many of these methods are also translatable to human research—and in some cases were originally developed for studying human disorders. For a comprehensive comparison of experimental human, animal and observational/field studies, the reader is referred to a recent review on this subject [42]. 

### 4.1. Visual Inspection of Actograms

The most obvious way of assessing circadian disruption is to simply look at experimental actograms. This visual inspection should always take the form of circadian disruption into account, and whether this is environmental or genetic in origin. This will also determine the appropriate controls, be they baseline conditions or wildtype littermates, respectively. Furthermore, when studying circadian disruption under different lighting paradigms, comparing activity relative to the external LD cycle is critical. This initial visual inspection of actogram data will guide the choice of which additional measurements are likely to be most informative. 

#### 4.1.1. Measurement

The initial visual assessment of actograms should take several different issues into consideration. How was activity measured? Running wheels give clear activity onsets, which are ideal for measuring free running period. However, under disruptive LD cycles or in disease-relevant models, running wheel behaviour may be affected, which can affect the resulting circadian data obtained. Was activity entrained or free running? If entrained, what is the phase relationship between activity onset and environmental zeitgebers? Is activity consolidated or fragmented? How long are animals active/inactive? It is recommended that hypotheses and predictions relating to specific parameters are made in advance of studying circadian behaviour, rather than an entirely hypothesis-free screening approach. Otherwise, if a large number of different circadian parameters are assessed, multiple test corrections should be applied to avoid false positives. 

#### 4.1.2. Strengths and Limitations

The strength of visual assessment of actogram data is that our visual systems are very good at identifying patterns in data. Critically, this initial assessment takes the experimental paradigm into account, as well as differentiating between changes in other metrics that arise for different reasons. For example, reduced amplitude rhythms could occur due to fragmentation, arrhythmicity or increased light phase activity. Visual assessment can also account for any artefacts or known errors in the data, such as breaks in data caused by technical problems during recording. The primary limitation of visual inspection of actograms is that it is very subjective, and we may see patterns in data where they do not really exist. As such, this approach is very dependent upon the experience and potential bias of the observer. Despite these limitations, the visual inspection of actograms should always be the starting point of any analysis of circadian disruption, and will guide the subsequent use of additional metrics. 

### 4.2. Periodogram Power 

The power of a periodogram provides a measure of the strength and regularity of the underlying rhythm, with higher values indicating robust rhythms. In circadian disruption—where rhythms are typically less robust—periodogram power is expected to be reduced and low values may indicate the absence of a significant circadian rhythm. The power of the chi-square periodogram (Q_P_) has been widely used as a measure of the robustness of circadian rhythms, and can be traced back to studies on the effects of constant light on the strength of activity and sleep rhythms in rats [49]. Analysis of Q_P_ based upon simulated and empirical data sets has shown that this provides a valuable measure of the robustness of circadian rhythms [50]. Periodogram analysis is particularly informative in internal desynchrony, where the power of multiple periodicities within a dataset will be evident.

#### 4.2.1. Measurement

The power of the chi-square periodogram (*Q_P_*) is the most widely used measure of periodogram power used to estimate robustness of circadian rhythms. Effectively, *Q_P_* is a ratio of the variance observed at a specific period compared against the total variance in the data set. For a dataset with N individual values (*X_i_* for *i* = 1 to N) which can be broken down into *K* blocks of period *P*, *Q_P_* is calculated as:

(1) $$Q_{P}=\frac{KN\sum_{h=1}^{P}{\left({\bar{X}}_{h}-\bar{X}\right)}^{2}}{\sum_{i=1}^{N}{\left(X_{i}-\bar{X}\right)}^{2}}$$

where *K* is the number of rows (‘days’) in *P* columns (measurements per ‘day’) based upon the period being tested. ${\bar{X}}_{h}$ is the mean of *K* values under each time unit measurement; $\bar{X}$ is the mean of all N values. Periodograms are then constructed by calculating *Q_P_* values for all periods within the desired range (e.g., 16 to 32 h). *Q_P_* has a probability distribution of χ^2^ with *P* − 1 degrees of freedom [18,50].

#### 4.2.2. Strengths and Limitations

Q_P_ is a standard output from most circadian analysis packages that estimate circadian period using the chi-square periodogram, such as ClockLab (Actimetrics Inc., Evanston, IL, USA), Circadian Rhythm Lab (www.circadian.org/periodogram.html), Actogram J [51], El Temps (www.el-temps.com), and BioDare [15]. As it is based on the chi-square periodogram it can be derived under both entrained and free-running conditions, although it requires regular data without missing values. Q_P_ values increase with the amplitude of the rhythm (between peak and trough) and are lower if the amplitude varies from day to day, for example if a rhythm is damping [21]. As the period of a rhythm is affected by environmental zeitgebers, higher Q_P_ values will be observed under entrained conditions. Finally, as Q_P_ values depend upon the number of data points per cycle and the number of cycles over which they are derived, this makes the comparison of Q_P_ values between studies more difficult.

### 4.3. Activity Onset 

A hallmark of normal circadian function is that activity onset is consistent from day to day (Figure 1A). In most records, the onset of activity is typically a more precise phase marker than the offset of activity [52]. As such, the variability in activity onset across multiple days—either relative to the light/dark cycle (phase angle of entrainment) or under constant conditions—provides a useful metric to describe the precision of circadian rhythms. Phase angle of entrainment and the variability in activity onset have been widely used in the study of circadian entrainment [53,54]. Activity onset is particularly informative when studying circadian misalignment and chronodisruption, where the phasing of activity with regard to environmental zeitgebers is expected to differ. Higher variability in activity onset has also been described in *Bdr* mutant mice [47]. 

#### 4.3.1. Measurement

Whilst there are no universal standards for defining activity onset, this is typically based upon the time point at which activity first exceeds a defined threshold. This can be any activity (>0), or the mean or median activity or a defined percentile of activity. For example, the commonly used ClockLab analysis package determines the 20th percentile of overall activity (of non-zero counts). ClockLab then converts the data into −1 for data points below this value, or 1 for data points exceeding this value. This threshold record is then convolved with a template consisting of N hours of −1 s and M hours of 1 s, with the default of N = 6 h, M = 6 h (corresponding to 6 h of inactivity followed by 6 h of activity). The onset of activity for each day is then placed at the maximum of this convolution (Actimetrics Inc., Evanston, IL, USA). This form of additional processing is valuable for reliably determining activity onsets, which can be prone to noise if based upon thresholding alone.

#### 4.3.2. Strengths and Limitations

Activity onset is readily determined by most circadian analysis packages (see examples in Section 4.2.2. above). However, the methods used to derive activity onsets may differ, which can influence comparisons between data sets. A further limitation of activity onset is that it is strongly influenced by the method used to measure activity. For example, running wheels give clear well-defined activity onsets with high signal to noise which are ideal for determining activity onset [8]. By contrast, methods based upon overall home cage movement, such as passive infrared sensors, may make activity onsets more difficult to accurately determine [16]. Under entrained conditions zeitgeber strength may also influence activity onset, and this has been shown to vary with light intensity [55]. 

### 4.4. Light Phase Activity

In nocturnal species, such as laboratory mice, activity is normally confined to the dark phase of the light/dark cycle [9,56]. A hallmark of disrupted rhythms is therefore an increased activity in the normally inactive light phase, and such changes are expected to occur in both circadian misalignment and chronodisruption. In diurnal species, dark phase activity can similarly be used to quantify the amount of activity occurring in the normal inactive phase. Light phase activity has been widely used to study defects in circadian entrainment to light as well as in disease-relevant models. Increased light phase activity has been described in both *Bdr* and *Grm2/3* knockout mice [47,48]. In addition, similar changes have been described in neurokinin 1 receptor (NK1R) knockout mice [57] and alpha synuclein (SNCA) mutant mice [58], which are mouse models relevant to attention deficit hyperactivity disorder and Parkinson’s disease, respectively. Light phase activity also provides an ideal measure to assess the impact of misaligned feeding [59], a specific example of circadian misalignment.

#### 4.4.1. Measurement

Light phase activity is simply expressed as a percentage of total daily activity:

(2) $$\frac{L}{L+D}$$

where L is the total activity in the light phase across the time course, and D is the total activity in the dark phase. Light phase activity may occasionally be expressed as a percentage of the total daily activity across the light phase to provide further temporal information [57].

#### 4.4.2. Strengths and Limitations

The main advantage of light phase activity is that it is very easily determined. However, it is perhaps most reliable under normal light/dark housing conditions, as it may be influenced by light intensity, photoperiod and other entrainment parameters, such as food availability [59]. Clearly, it is also not possible to use light phase activity under free-running conditions. The measurement of light phase activity is very dependent upon the method of monitoring. As running wheels only record voluntary activity, light phase activity is typically extremely low. By contrast, passive infrared sensors will detect small home cage movements. Whilst often considered ‘noise’, this can provide useful information regarding general activity during the light phase [8].

### 4.5. Activity Bouts

As a result of less consolidated activity and rest phases, circadian disruption is associated with an increased number and reduced duration of activity bouts. The number and duration of activity bouts are frequently used as a measure of fragmentation in circadian disruption. Due to the inappropriate phasing of activity/rest cycles with regard to the external environmental, circadian misalignment, internal desynchrony and chronodisruption are all expected to affect activity bouts.

#### 4.5.1. Measurement

Bouts are typically expressed as bouts per day and average bout duration. However, a key issue with the analysis of activity bouts is the definition of an activity bout used. Bouts are typically defined as a period during which activity does not fall below a specified threshold. For example, ClockLab (Actimetrics, Evanston, IL, USA) uses a definition of ‘the period during which the activity never falls below N counts per minute for longer than M minutes at a time, where N is the Bout threshold, and M is the Maximum Gap Length’. However, as N and M are variable, the definition of a bout is open to interpretation. Finally, it should be noted that Fourier analysis can also be used to detect ultradian bouts in circadian data, in addition to its application for determining period. 

#### 4.5.2. Strengths and Limitations

Whilst the number and duration of activity bouts provide an obvious measure of the fragmentation of activity rhythms, the results of any such analysis will depend upon the criteria used to define a bout. Activity bouts also show an obvious circadian dependence, and in nocturnal rodents most activity will occur during the active dark period. As such, bout analysis may express bouts during the light and dark phases, or even across the 24 h cycle [47]. As non-human species typically show less consolidated activity and rest in comparison to humans, bout duration may be biased by the high number of short bouts. To address this issue, some studies have compared activity bouts across multiple different durations e.g., 0–1 min, 1–10 min, 10–60 min, etc.; [60]. Finally, it should be noted that the ability to accurately assess bout length is limited by sampling frequency. 

### 4.6. Inter-Daily Stability

Inter-daily stability (IS) measures the day-to-day reproducibility of rest/activity cycles. Patterns of activity are typically reproducible in healthy individuals, whereas circadian disruption results in more variable rhythms. IS was first described in the study of elderly human patients with Alzheimer’s disease [61]. It has subsequently been widely used in a number of human studies [42,62,63], and has also been adopted for studying circadian disruption in animal models. Due to the day-to-day changes in the relationship between circadian and environmental time, decreased IS may be expected in internal desynchrony. However, circadian misalignment and chronodisruption may not influence IS if a stable new phase-relationship is established. Genetic examples of changes in IS are provided by *Bdr* mutant mice and *Grm2/3* knockout mice, which both show decreased IS compared to wildtype controls [47,48].

#### 4.6.1. Measurement

IS is based upon the 24 h value from the chi-square periodogram, normalised for the number of data. It provides a measure of the strength of coupling between the rest/activity rhythm and zeitgebers. IS is essentially the variance of the average 24 h pattern of activity expressed as a ratio of the total variance.

(3) $$IS=\frac{N\sum_{h=1}^{p}{\left({\bar{X}}_{h}-\bar{X}\right)}^{2}}{p\sum_{i=1}^{N}{\left(X_{i}-\bar{X}\right)}^{2}}$$

where *N* is the number of data points; *p* is the number of data per day (24 if data are in 1 h bins); $\bar{X}$ is the mean of all data; ${\bar{X}}_{h}$ are the hourly means and $X_{i}$ are the individual data points. This provides a ratio between 0 (no reproducibility) and 1 (perfect day-to-day reproducibility). The relationship between *Q_P_* and *IS* is clear from the similarity between Equations (1) and (3), which only differ in that the latter excludes the number of cycles (*K*) of the specific period (*P*), and corrects for the number of data per day (*p*). 

#### 4.6.2. Strengths and Limitations

Whilst relatively simple to calculate, due to its origins in the study of human circadian rhythms, many analysis packages designed for animal research do not calculate IS. As IS is a ratio of variance between 0 and 1, it avoids many of the problems associated with comparing Q_P_ values on different time scales. However, as a single index of day-to-day reproducibility IS does not provide information relating to how rhythms are disrupted. Whilst it is possible to determine IS under non-24 conditions, this is complicated by the modification of the calculations for circadian period.

### 4.7. Intra-Daily Variability

Intra-daily variability (IV) is a measure of the fragmentation of activity rhythms. First introduced for the study of patients with Alzheimer’s disease [61,62,63], like IS it has been readily adopted for the study of animal models of circadian disruption. IV measures the frequency of transitions between activity and rest across the day. More transitions between activity and rest result in higher IV scores. As with activity bouts, circadian misalignment, internal desynchrony and chronodisruption may all increase IV due to the inappropriate phasing of activity/rest cycles with regard to the external environment. A good example of this occurs in aging, where IV increases steadily with age in mice [64].

#### 4.7.1. Measurement

IV is calculated the summated variance between consecutive hours (first derivative) expressed as a ratio of the total variance.

(4) $$IV=\frac{N\sum_{i=2}^{N}{\left(X_{i}-X_{i-1}\right)}^{2}}{\left(N-1\right)\sum_{i=1}^{N}{\left(\bar{X}-X_{i}\right)}^{2}}$$

where *N* is the total number of data; $X_{i}$ represent individual data points and $\bar{X}$ is the mean of all data. It should be noted that unlike IS, IV is not constrained to values between 0 and 1, and values over 1 are possible.

#### 4.7.2. Strengths and Limitations

IV provides a useful measure of fragmentation of activity and rest. Similar to IS, due to its origin in the study of human rhythms, IV is less likely to be a standard output of many standard laboratory animal analysis packages. It should be noted that IV is based upon the hour-to-hour transitions, reflecting relatively long periods of activity and inactivity. This was originally intended to detect daytime napping or night-time waking in human subjects, avoiding frequent transitions associated with daytime activities [62]. However, when IV is applied to the study of circadian disruption in animal models, this may overlook biologically relevant changes.

### 4.8. Relative Amplitude 

Perhaps the most obvious measure of the strength of any biological rhythm is its amplitude. A simple metric that has been widely used in human studies is relative amplitude (RA), again originating from studies in patients with Alzheimer’s disease [61,63]. RA is a simple measure of the difference between periods of peak activity and rest. As healthy rhythms are assumed to display consolidated activity and rest periods, RA is expected to be reduced when normal circadian rhythms are disrupted. Both circadian misalignment and chronodisruption will reduce RA, with internal desynchrony potentially resulting in RA values that fluctuate as periods move in and out of phase.

#### 4.8.1. Measurement

Relative amplitude is based upon the difference between the period of maximum and minimum activity across the 24 h cycle. In humans, to reduce the influence of noise, the most active 10 h and least active 5 h are used.

(5) $$RA=\frac{M10-L5}{M10+L5}$$

where M10 is the most active 10 h period and L5 is the least active 5 h period in the average 24 h pattern [61,63]. 

#### 4.8.2. Strengths and Limitations

RA provides a simple measure of rhythm amplitude. However, as this ratio is based upon the average 24 h pattern, it does not account for the variability in activity and rest across multiple days. As it is based upon periods of consecutive maximum activity (M10) and minimum activity (L5), any fragmentation of rhythms will influence RA. Perhaps the greatest limitation of RA for the study of circadian disruption in animal models is that the definitions of M10 and L5 are based upon consolidated periods of activity and rest, which do not reflect the activity patterns of many non-human species, which are typically much less consolidated. As such, unlike IS and IV, RA has not been widely used in studies of circadian disruption in animal models, and where it has been used this may be dependent upon different definitions of maximum and minimum activity.

## 5. Additional Considerations and Alternative Approaches

Despite the use of a wide range of different metrics in the literature, there is no single standard for measuring circadian disruption. As a result, in many studies multiple different measures are used. For example, in the *blind-drunk* (*Bdr*) mutant mouse, periodogram power, variability in activity onset, light phase activity, activity bouts, interdaily stability and intradaily variability were all shown to be disrupted [47]. However, it should be noted that of the different measures of circadian disrupted described above are not independent. To illustrate this, we took published data from 24 male C57BL/6 mice housed for 7 days under a 12:12 light:dark cycle [16]. Periodogram power, variability in activity onset, light phase activity, activity bouts, interdaily stability, intradaily variability and relative amplitude were calculated from this dataset to provide normal ranges for these parameters under entrained conditions (Table 1). In addition, the Pearson’s correlation coefficient between these measures was also determined across this dataset (Table 2), demonstrating the inter-related nature of these measures. For example, as interdaily stability is derived from Q_P_ normalised to the number of data, it is not surprising that interdaily stability is positively correlated with Q_P_ (0.81). As higher periodogram power is associated with greater amplitude rhythms, Q_P_ also shows a strong positive correlation with relative amplitude (0.74) and a negative correlation with light phase activity (−0.74) and interdaily variability (−0.81)—both of which are associated with low amplitude rhythms. High light phase activity will result in a greater L5 value, which is used to determine relative amplitude. This is evident in the strong negative correlation between light phase activity and relative amplitude (−0.90). As a result of their inter-relatedness, highly disrupted circadian rhythms are likely to show significant differences in many if not all of these measures. By contrast, caution should be exercised where only a single parameter is affected, as this may represent a false positive. To date, the performance of these different measures for detecting circadian disruption in different datasets has not been well-explored. The use of well-characterised or simulated datasets provides an ideal opportunity to investigate the strengths and limitations of these methods in more detail.

A key feature of a circadian process is that it occurs with a daily period. This assumption that period and amplitude are fixed is termed ‘stationarity’ [50]. However, it is becoming increasingly apparent that biological time series often demonstrate changes in period and amplitude, which has led to an increasing interest in ‘non-stationary’ methods such as wavelet analysis. Wavelet methods can be effective in removing trends and noise from time series data, as well as uncovering ultradian components [65,66,67]. A key feature of circadian disruption is that the amplitude and day-to-day reproducibility of rhythms is impaired, as measured by parameters such as Q_P_, light phase activity, IS and relative amplitude. To date, wavelet analysis has not been widely used to study circadian disruption in animal models. However, this approach may provide valuable insights into the dynamic changes that occur over time. Similarly, the adaptation of some of the methods described above using a rolling analysis window may enable changes in circadian disruption over time to be studied. Finally, alternative approaches are continuously being developed, which may provide additional information regarding the changes in physiology and behaviour that occur during circadian disruption. For example, studies combining temperature (T), activity (A) and body position (P) data into a single parameter (TAP) have been developed for studying human circadian disruption. This study also combined IS, IV and RA to provide a single Circadian Function Index (CFI), which provided a measure of rhythm robustness [68]. The use of such alternative metrics for characterising circadian function are likely to help provide a better description of the exact nature of circadian disruption in different conditions. 

## 6. Conclusions

Circadian rhythms are a hallmark of normal healthy physiology, and increasing evidence suggests that these rhythms are disrupted in a wide range of disorders. Under such conditions the circadian system retains its normal period of around 24 h, but rhythms become less robust and more fragmented. This poses a quantitative problem of how to measure a poor rhythmic process. Many of the analytical tools developed to measure circadian rhythms are based upon defining circadian period or activity onset. Whilst these methods are good at detecting the presence or absence of rhythmic processes, they are often not suited for detecting disrupted rhythms. As a result, a wide range of different parameters have been used to study circadian disruption. Here we describe how these measures have been applied to the study of animal models, how they are calculated as well as their strengths and limitations. We go on to discuss how these measures are inter-related as well as alternative metrics that may improve our ability to characterise circadian disruption in more detail. Improved quantification of disrupted rhythms is essential if we are to assess the efficacy of new therapeutic interventions to help treat the increasingly widespread problem of circadian disruption.

## Figures and Tables

**Table 1:** Normal ranges of circadian parameters in mice.

Parameter	Mean ± SD	Range	Notes
Periodogram power (Q_P_) ^1^	22,982 ± 5686	13,859–35,821	Chi-square periodogram
Activity onset ^2^	3.33 ± 2.22	0.00–6.41	Based on bouts > mean
Light phase activity (Light%)	14% ± 4%	5–22%	
Activity bouts (bouts/day)	278 ± 63	162–420	Based on sensitive PIR
Interdaily stability (IS)	0.73 ± 0.09	0.47–0.88	
Intradaily variability (IV)	1.18 ± 0.32	0.67–1.90	
Relative amplitude (RA)	0.80 ± 0.07	0.66–0.94	Based on M10 and L5

See Section 5 for additional information. ^1^ Q_P_ is dependent upon the sampling rate of measurement (these values are based on 10 s); ^2^ Expressed as standard deviation over 7 days.

**Table 2:** Interrelation between different circadian disruption parameters.

	Q_P_	Onset	Light%	Bouts	IS	IV	RA
**Q_P_**	1.00	−0.46	−0.69	−0.45	0.81	−0.81	0.74
**Onset**		1.00	0.60	0.33	−0.31	0.31	−0.47
**Light%**			1.00	0.20	−0.70	0.59	−0.90
**Bouts**				1.00	−0.20	0.46	−0.28
**IS**					1.00	−0.79	0.73
**IV**						1.00	−0.72
**RA**							1.00
